# Restoration of Calmodulin-Like Skin Protein as Treatment for Alzheimer's Disease

**DOI:** 10.3389/fneur.2021.771856

**Published:** 2021-10-25

**Authors:** Masaaki Matsuoka

**Affiliations:** Department of Pharmacology, Tokyo Medical University, Tokyo, Japan

**Keywords:** Alzheimer's disease, calmodulin-like skin protein, adiponectin, defense factor, CLSPCOL

## Introduction

The neurotoxicity linked to pathogenesis may be generated by the cumulative effect by multiple biological insults. Therefore, the removal of only one of the insults may not affect the disease status sufficiently. Apart from the increase in neurotoxicity, it is possible that a reduction of a neurotoxicity-suppressing factor may be essential for the onset of the disease. Theoretically, the disease may not begin even in the presence of enough levels of neurotoxic insults, if the neurotoxicity-suppressing factor blocks the damage to the neurons, preventing the emergence of neurotoxicity.

## Current Status of Alzheimer's Disease-Modifying Therapy

Alzheimer's disease (AD) is the most prevalent dementia-causing neurodegenerative disease. It has been hypothesized that amyloid β (Aβ) and/or intraneuronal aggregated hyperphosphorylated tau are the central insults responsible for the AD pathogenesis (1–5). Based on this hypothesis, multiple therapeutic agents against amyloid β (Aβ) and/or intraneuronal aggregated tau have been developed to block disease progression. However, contrary to the expectation, clinical trials for such drugs have not proven their efficacy (6–8). Exceptionally, high-dose monoclonal antibody against Aβ named aducanumab have been proven to be effective in an uncertain manner (9–11). One of two phase III clinical trials for aducanumab showed that the administration of it retarded disease progression mildly whereas the other did not. It was conditionally approved by the U.S. Food and Drug Administration in June, 2021.

Several scientists have insisted that this setback in the development of anti-Aβ drugs may be due to late administration of the drugs, speculating that most AD patients seem to have a long history (more than 20 years) of Aβ accumulation in the brain at the time of AD diagnosis (12, 13). Therefore, it is assumed that neurons in such patients would be already irreversibly damaged when AD was first diagnosed and continued to die even after the neurotoxic insults were successfully removed. Another possible reason for the lack of drug efficacy may be that multiple insults other than Aβ and aggregated tau cause neurotoxicity and the removal of only Aβ or aggregated tau is not enough to halt the disease progression. This interpretation is supported by a growing number of studies that show the presence of the multiple neurotoxic insults for AD other than Aβ and aggregated tau (14–17).

## Restoration of a Reduced Neurotoxicity-Suppressing Factor For AD

Given the complicated and partially proven AD-linked neurotoxic insults, an alternative strategy may be necessary for the AD treatment. Restoration of a reduced a neurotoxicity-suppressing factor could be a reasonable strategy for the AD treatment if the reduction of the neurotoxicity-suppressing factor is essential for the disease onset.

Calmodulin-like skin protein (CLSP), a secretory peptide, inhibits neuronal death linked to AD *in vitro* (18) and the transgenic overexpression of the *CLSP* gene reverses hippocampal synaptic loss and memory impairment in a mouse line named APPswe/PSEN1dE9 mice that transgenically overexpressing two familial AD-causative genes, human amyloid precursor protein with the Swedish mutation and deletion of exon 9 of human presenilin-1 genes (19). The Aβ plaques are formed at their middle age when dementia begins in the mice. A recent study has shown that the CLSP activity is reduced in AD patients and the APPswe/PSEN1dE9 mice, and the restoration of the CLSP activity reverses memory impairment and hippocampal synaptic loss in the APPswe/PSEN1dE9 mice (20). Thus, it is likely that the restoration of the CLSP activity may provide a reasonable and effective therapeutic strategy for AD.

CLSP was originally discovered as an agonist of the heterotrimeric humanin receptor. CLSP, mainly produced in skin tissues, is transported into the central nervous systems where it may suppress AD-linked neurotoxicity (14). Although there are sufficient levels of CLSP in the central nervous systems of AD patients (21), larger amounts of multiple CLSP inhibitors appear to suppress the CLSP activity completely (20). However, even in the presence of larger amounts of CLSP inhibitors, adiponectin binds to CLSP and protects CLSP from the inhibition by CLSP inhibitors in a non-competitive manner. Thus, the level of adiponectin determines the CLSP activity in the central nervous system. We have found that the levels of adiponectin and the CLSP activity are diminished in the central nervous systems of human AD patients and the APPswe/PSEN1dE9 mice (20). These results suggest that the insufficiency in the CLSP activity is essential for the onset of AD. To further prove this, it was shown that the restoration of the reduced CLSP activity corrected memory impairment and hippocampal neurosynaptic loss in the APPswe/PSEN1dE9 mice (20).

To recover the reduced CLSP activity, a hybrid peptide named CLSPCOL has been developed (20). CLSPCOL is composed of CLSP(1–61) and the collagen domain of adiponectin (COL). CLSP(1–61) comprises the full cell-death-suppressing activity *via* the heterotrimeric humanin receptor and is deficient in the C-terminal domain of CLSP to which the CLSP inhibitors bind. Similar to adiponectin, COL protects CLSP from the suppression by CLSP inhibitors and potentiates the CLSP activity. However, COL does not seem to bind to and activate the canonical adiponectin receptors. This hybrid peptide is non-immunogenic and does not cause apparent toxicity in mice. Moreover, it is efficiently recruited to the central nervous system. A CLSPCOL molecule is also thought to bind to and activate another CLSPCOL molecules and the endogenous CLSP. The half-life of CLSPCOL in the central nervous system appears to be more than 24 h. The CLSPCOL therapy that had been administered for 7 days improved the cognitive function of the advanced-phase APPswe/PSEN1dE9 mice (20), suggesting that the effect of this therapy may emerge very quickly. These characteristics of the peptide favor the potential of CLSPCOL as a drug. However, it remains unknown until systematic pharmacokinetics and toxicity tests will be performed whether it can go to the clinical tests.

## Discussion

Adiponectin is a rate-limiting regulator of the CLSP activity. Accumulating evidence supports the link between adiponectin and the AD pathogenesis. Multiple reports have shown that the reduction of adiponectin in the central nervous system is linked to the onset of AD. Two previous studies showed that the levels of adiponectin, are downregulated in the central nervous systems of AD patients (20, 22). It was also shown that the levels of SH3BP5, the intraneuronal marker and effector of the CLSP signal, were diminished in the brains of AD patients (20). Adiponectin-reducing polymorphisms in the *adiponectin* gene enhance the onset of AD (23). Knockout of the *adiponecti*n gene causes AD-like pathology in mice (24).

The strong points of the CLSPCOL therapy are as follows. First, it appears to be effective against all AD-relevant neurotoxixities. Sufficient levels of CLSP activity can suppress all types of neurotoxicity linked to AD in both *in vitro* and *in vivo* conditions, even in the presence of overwhelming neurotoxic insults (14, 18–20). In reality, *in vitro*, analogs of humanin, an endogenous agonist of the heterotrimeric humanin receptor, suppress all kinds of AD-related neuronal death, induced by high concentrations of Aβ and ectopically expressed familial AD-causative mutants of amyloid-β precursor protein and presenilin 1 and 2 [(14) for review]. Second, it appears to exhibit neurotoxicity-suppressing effect without affecting Aβ levels both *in vitro* and *in vivo* [(14, 19)]. Unfortunately, there are no animal models mimicking neuronal loss or death of human AD. They only mimic Aβ plaques and Aβ-induced synaptic loss of neurons. Therefore, it is currently impossible to examine whether the CLSPCOL therapy is effective against neuronal loss or death *in vivo* that is assumed to be directly linked to severe cognitive impairment of human AD cases.

The exponentially growing number of AD patients indicates that AD has become a serious threat to the elderly in our society. In this regard, the CLSP restoration may be the next promising strategy for the treatment of AD.

## Author Contributions

The author confirms being the sole contributor of this work and has approved it for publication.

## Conflict of Interest

The author declares that the research was conducted in the absence of any commercial or financial relationships that could be construed as a potential conflict of interest.

## Publisher's Note

All claims expressed in this article are solely those of the authors and do not necessarily represent those of their affiliated organizations, or those of the publisher, the editors and the reviewers. Any product that may be evaluated in this article, or claim that may be made by its manufacturer, is not guaranteed or endorsed by the publisher.
